# Costing the scaling-up of human resources for health: lessons from Mozambique and Guinea Bissau

**DOI:** 10.1186/1478-4491-8-14

**Published:** 2010-06-25

**Authors:** Amanda K Tyrrell, Giuliano Russo, Gilles Dussault, Paulo Ferrinho

**Affiliations:** 1 World Health Organization Representative Office, 63 Tran Hung Dao Street Hanoi 10000, PO Box 52, Viet Nam; 2 Department of International Health, Instituto de Higiene e Medicina Tropical, Rua da Junqueira 100, Lisbon, 1349-0008 Portugal

## Abstract

**Introduction:**

In the context of the current human resources for health (HRH) crisis, the need for comprehensive Human Resources Development Plans (HRDP) is acute, especially in resource-scarce sub-Saharan African countries. However, the financial implications of such plans rarely receive due consideration, despite the availability of much advice and examples in the literature on how to conduct HRDP costing. Global initiatives have also been launched recently to standardise costing methodologies and respective tools.

**Methods:**

This paper reports on two separate experiences of HRDP costing in Mozambique and Guinea Bissau, with the objective to provide an insight into the practice of costing exercises in information-poor settings, as well as to contribute to the existing debate on HRH costing methodologies. The study adopts a case-study approach to analyse the methodologies developed in the two countries, their contexts, policy processes and actors involved.

**Results:**

From the analysis of the two cases, it emerged that the costing exercises represented an important driver of the HRDP elaboration, which lent credibility to the process, and provided a financial framework within which HRH policies could be discussed. In both cases, bottom-up and country-specific methods were designed to overcome the countries' lack of cost and financing data, as well as to interpret their financial systems. Such an approach also allowed the costing exercises to feed directly into the national planning and budgeting process.

**Conclusions:**

The authors conclude that bottom-up and country-specific costing methodologies have the potential to serve adequately the multi-faceted purpose of the exercise. It is recognised that standardised tools and methodologies may help reduce local governments' dependency on foreign expertise to conduct the HRDP costing and facilitate regional and international comparisons. However, adopting pre-defined and insufficiently flexible tools may undermine the credibility of the costing exercise, and reduce the space for policy negotiation opportunities within the HRDP elaboration process.

## Introduction

It is widely accepted that a dramatic expansion of the health workforce worldwide, and in particular for sub-Saharan Africa, is necessary to achieve universal coverage by health services. What remains problematic is how this expansion can be achieved in countries with limited financial resources and inadequate training capacity.

This paper presents and discusses two separate human resources for health (HRH) costing exercises carried out in the context of the elaboration of national human resources development strategies. The paper's objective is to present and analyze two separate examples on the elaboration of costing exercises in information-poor settings, and illustrate how the lack of HRH financial and cost data was overcome in Mozambique and Guinea Bissau. The paper's ultimate goal is to contribute to the debate on costing tools and strategies, and more specifically to identify advantages and disadvantages of certain key methodological aspects. In doing so, it highlights the importance of adapting any costing methodology to specific country needs and contexts.

HRH costing methodologies recommended in the literature are briefly reviewed in the first part of the document, followed by a description of the case study methodology used to compare the Mozambican and Guinean Bissau costing of the national scaling-up of the health workforce. The context, process and actors involved in the Mozambique and Guinea Bissau case studies are then presented along with the results of the costing exercises. The discussion highlights the lessons learned and stresses the advantages of selecting country-specific costing tools.

### Literature review

Martineau et al (2008) highlight the importance for low-income countries to develop a clear strategy to develop their health workforce, including a projection of costs over a multi-year period [1]. A 'costed' plan is credited with helping identify financing gaps and serving as an advocacy tool in the process of mobilizing resources. The classical approach to planning and costing Human Resources Development Plans (HRDP) in the health sector involves four steps: (a) estimating the numbers of additional staff needed; (b) multiplying those numbers by current and future public sector salaries and allowances; (c) calculating financial needs on the basis of projected available funds, and (d) discussing financing options [2]. However, this 'rational' approach does not take into account the dynamics of the labour market, and often ignores health personnel's propensity to emigrate. Bloor and Maynard consider it important to factor in the supply and demand of health personnel in the public and private sector [3]. Rutten argues that HRH planning should adopt general equilibrium models bringing together labour market factors and funds availability [4].

The World Health Organization (WHO) has recognised the importance of the issue, and launched initiatives such as the Working Group on Tools and Guidelines to strengthen the analysis of the costs of HRH development [5], and to propose guidelines. The Global Health Workforce Alliance (GHWA) supported the development of a Resource Requirements Tool (RRT) to help local governments estimate the costs of HRH plans and policies [6]. The RRT provides a methodology to collect information on HRH and on the local economy, including labour markets, and the tool is organised in an Excel-based file divided into data-entry, scenario and output sheets. By initially taking the calculated HR requirements from national HRH plans, the tool helps calculate and project the costs of different HRH training and recruitment options available to local Governments [7]. The tool calculates salary and incentives (financial, as well as the monetary value of non-financial incentives, such as free meals, transport, and accommodation), as well as initial training, capital and equipment expenses, and compares them with the projected funds availability. The concept of 'fiscal space', i.e. a country's ability to find the financial resources required to finance the anticipated spending[8], - is evaluated by taking into account the country's expected economic growth, the current level of GDP, the share of Government resources allocated to health, and the share of health spending devoted to personnel [9]. The RRT also takes into account the number of HRH employed in the private sector, but not consumers' willingness to pay for health services, nor the possible interactions between public and private providers.

Recent work shows that many National HRH Strategies fail to include a comprehensive review of costs: this is the case for Eritrea, Rwanda, Sierra Leone, Zambia, South Africa and Sudan [10,11]. In fact, there are few documented examples of costing such strategies in low-income countries. In the following sections, the paper describes the methodologies used and the lessons learned from two separate case studies in Mozambique and Guinea Bissau, and tries to relate them with the theoretical work currently being developed on the subject.

## Methods

Two separate costing exercises were conducted in Mozambique [12] and Guinea Bissau [13] between 2007 and 2009, at the request of the respective health authorities. For the two exercises, the authors developed distinct, context-specific methodologies taking into account the countries' financial reporting systems and availability of cost information. The description of how such exercises were conducted, and of the resulting costing methodologies that were developed, constitutes the basis of the present paper.

A descriptive multi-case study approach [14] was selected to compare the two experiences and answer the following questions: (a) what to do and how to do it when undertaking HRH plans costing; and (b) what conditions need to be in place to enable a successful costing exercise. The two case studies were analysed and compared, and lessons were extracted from the two distinct exercises focusing on the context, on the elaboration process, on the actors involved, on the methodological approach, and on the specific problems that each methodology had to overcome. A framework was adapted from Walt and Gilson [15] to analyse the following methodological and policy-making dimensions from the two cases: (a) the methodologies developed for HRH costing; (b) policy contexts and environments; (c) policy processes; and (d) actors involved. A recent review (Gilson et al. 2008) concluded that such an approach is effective to analyze diverse policy initiatives in different contexts [16,17].

The costing studies were conducted independently in the two Portuguese-speaking countries, in collaboration with the international and national teams responsible for the development of HRH plans. Information was collected through interviews with policy makers, health managers, and other relevant stakeholders, as well as through documental analysis. Sources of information used were: (a) human resources projections where available; (b) official salary tables and any additional relevant documentation on salaries' policy; (c) HRH training programme funding data; and (d) domestic budget and external aid data.

## Results

### Costing HRH projections in Mozambique

#### Context, process and actors involved

Since the end of the war in 1992, Mozambique has progressively been rebuilding its health network, through reconstruction and rehabilitation of physical infrastructures, as well as training and recruitment of its health workforce. The country is still characterized by a high burden of disease, and faces the challenge of an unreformed health system, including a severe deficit of HRH in all categories of workers [18]. The elaboration of Mozambique's health sector's Human Resources Development Plan (HRDP) 2008-15 was developed in the context of strong political commitment from the Government to alleviate poverty and to achieve the Millennium Development Goals (MDGs).

At the time of elaboration of the plan (2007-2008), the Government of Mozambique was engaged in the process of designing a new salaries policy for public servants, with a potentially significant impact on the wage bill across sectors. Donors financed a number of salary top ups, incentives and a limited number of salaries through a common funding mechanism (PROSAUDE, that is, the Health Sector-Wide Approach fund), as well as direct financing to provincial authorities, but the government essentially financed the wage bill. The development of the HRDP aimed at increasing access and quality of services to the population, particularly in remote and disadvantaged areas. Both the Ministry of Health and development partners were committed to the process, and considered the costing exercise an integral part of the plan, as the two were developed in concurrence by the same team of consultants. Financial implications of different scenarios, based on the number of workers to be trained and recruited and on varying salary levels, served as a basis for policy discussions and informed the final decisions on the plan's proposals.

The main objectives of the costing exercise were: (a) to analyse current levels of expenditure on HRH, both from internal and external sources; (b) to calculate the real cost of training in the health sector, including initial and on-the job training (or continuous medical education), post-graduate training and scholarships, and; (c) to calculate the cost of different scenarios of HRH development until 2015, based on the HRH strategy and other relevant existing policy documents (e.g. government salaries policy, incentives policy).

The costing exercise took place from late 2007 until mid-2008, and was supported by a team of external specialists (a senior human resources specialist, a statistician, and a health economist), who visited Mozambique on a number of occasions and for relatively long periods (two months). The focal point in the Ministry of Health (MoH) was the Human Resources Department. One key source of information was the National Health Institute, where detailed cost data was collected, analysed, and crosschecked with expenditure information at the MoH on other training institutions across the country, in order to calculate unit costs of training.

#### Methodology used in Mozambique

Cost projections were based in large part on the training outputs and salary increase targets already defined for the HRDP. Personnel projections took into account attrition rates due to deaths, retirement, and moves to the private sector, including to international organisations and NGOs. Macroeconomic data, such as annual inflation and exchange rates were based on the government's Mid-Term Expenditure Framework (MTEF) 2008-10. Where possible, a bottom up approach was adopted, which implied collecting cost data at the grassroots, and combining unit costs with input quantities in order to generate total costs. Where data were limited or unavailable, a step-down methodology was used, breaking down costs to the smallest possible denominator. Emphasis was put on ensuring that revisions of the projections could easily be made in the future, as new data became available or unforeseen changes affected the costs significantly. Future costs and expenditures were calculated in real and nominal terms according to the formula: Present value* (1+i)^n^.

Spending projections were calculated through a set of costing sheets in Excel to match the needs of the sector in terms of relevant cost components, classification of expenditure, and the structure and content of existing information, such as the government salary levels classification. Where relevant and possible, the sheets were interlinked through a set of simple formulae and references in order to ensure that any change in inputs (e.g. number of students) was reflected in outputs (e.g. annual health workforce). Besides HRH typical recurrent costs such as salary or training expenditures, capital expenditures were also included, as these were considered key to scaling-up the national health workforce. Capital investments were identified on the basis of information obtained from the infrastructure department of the MoH. They corresponded to the cost (estimated by MoH) to expand and rehabilitate the physical health network, including capital works (secondary care hospitals) and equipment (scanners, x-ray machines, etc.). Only the purchase cost of tangible assets used to strengthen the training network were estimated (e.g. building and equipping of training institutions); the exercise did not attempt to include an estimation of the accounting value of assets. It was also assumed that those assets with a life span longer than the study's expenditure projections were not going to need replacing. Maintenance costs for the health system as a whole were included in the broader costing of the health strategy and were taken into account in this section to avoid double counting.

**Figure 1:**
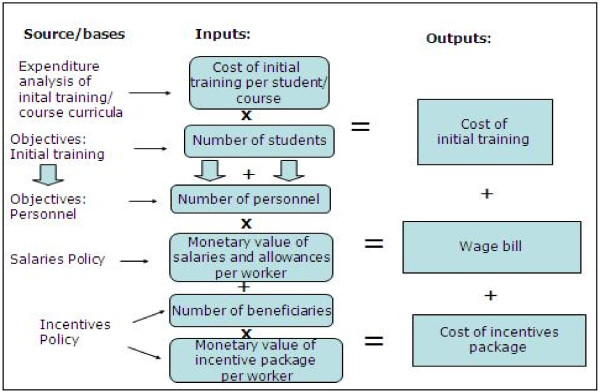
**Illustration of the rationale of the Mozambique HRH costing model**. Source: Tyrrell (2008) 12.

The set of detailed sheets was subsequently aggregated to present the summary of planned expenditure per area of HRH over the years. Figure 1 illustrates the rationale behind the costing model developed, and the table in Additional file 1 is the summary of cost components and sub-components identified and sources of information. Salaries and allowances (the wage bill) included workers' base salary and legal supplements, such as subsidies for working in remote areas, for management positions, working extra hours, etc. Additional incentives were considered to be those relating to civil servants also sponsored by donors. In the absence of a finalized incentives policy, the critical assumption was made that the value of incentives summed up at least 26% of the salary mass.

Training costs were divided into four sub-components, namely initial and on-the-job training, postgraduate training and scholarships. Recurrent and capital expenditure were considered where relevant. For initial training, in the absence of specific targets for the number of beneficiaries and number of courses, assumptions were made to derive the number of beneficiaries, on the basis of the analysis of courses offered to date and of the specialties to be prioritized. Other important components of cost of the HRDP were foreign doctors, Community Health Workers (CHW), technical assistance, and other associated expenditures (Figure 1).

**Figure 2:**
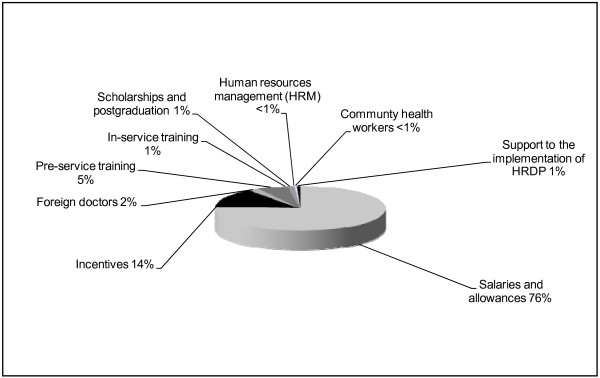
**Total projected HRH expenditure by component, Mozambique (2008-2015)**. Source: Tyrrell (2008) 12.

The total cost of the HRDP was estimated at approximately two billion USD over 8 years, of which over three quarters were planned to finance the wage bill, and another 14% the incentives package to support worker deployment, motivation and retention (Figure 2).

Although in relative terms the planned expenditure in other areas of HRH appears small, a total of over 100 million US dollars was estimated necessary for initial training between 2008-15, of which 28% for infrastructure and equipment; and 11 million US dollars were dedicated to on-the-job training. The estimated cost of foreign doctors amounted to 43 million, the annual value reducing considerably between 2008 and 2015, under the assumption that, as the country increases its capacity to train national doctors, there will be less of a need to contract foreign ones. In addition to costs directly associated with scaling up the health workforce between 2008 and 2015, it was estimated that almost one billion USD would be necessary for infrastructure and equipment, in order to enable complete timely implementation of the HRDP.

Three scenarios were developed to estimate HRH needs, varying the criteria used for personnel projections. Given the uncertainty regarding the salaries policy, a sensitivity analysis was performed, altering assumptions on salary increases for different levels and categories of workers, to reflect the different salary policy options in negotiation [19]. The scenarios varied from an average of 1410 graduates per year, producing the lowest number of workers by 2015 (39 638), and costing an average of 158 million USD annually, to 2036 graduated per year, at an average cost of 174 million USD. In the end, the government chose the most costly scenario, where the health workforce was increased by 44% (from 25 683 in 2006 to 45 654), based on staffing norms and on priority to workers in MDG-related areas.

The costing exercise uncovered that expenditure on HRH would have to increase from 50 million US dollars in 2006 to 400 million US dollars in 2015 in order to accommodate the training targets, increasing steadily over the years. The eight-fold increase in the cost foreseen over nine years is primarily due to the plan's stated objectives of rapidly scaling up the health workforce (at an average rate of over 2200 workers per year), while at the same time guaranteeing adequate remuneration to workers, particularly for those professionals operating in remote, more disadvantaged areas, and in priority specialties for the attainment of the MDGs.

Since its completion, data from the costing exercise have been used to update the costing of the Health Sector Strategic Plan and the Medium Term Expenditure Framework (MTEF). It also served to prepare the budget for the Health Systems Strengthening (HSS) component of Mozambique's HIV/AIDS proposal to Round 9 of GFATM, still awaiting grant signature.

### The HRH plan costing in Guinea Bissau

#### Context, process and actors involved

At the time of elaboration of its HRH plan, Guinea Bissau was going through a deep political and social crisis, which impacted significantly on HRH planning and management. Ferrinho (2009) estimated that without scaling-up training and recruitment, HRH were projected to decrease from 2010 onwards [20]. The fragility of the situation was exacerbated by the fact that, since independence, donors had exclusively, and to a very limited scale, supported HRH training, whereas the government paid the wage bill, usually with delays of many months.

Local capacity for the elaboration of the HRH development plan (HRDP), and in particular to conduct its costing, was low. No economic data existed on the cost of training, or on the overall level of external funds available to support it. The costing exercise was designed as an iterative process, and as part of the development of the main HRDP. Preliminary findings on budget ceilings and cost implications provided an input into the elaboration of the main HRDP, and the costing report was later adapted to incorporate the policy options selected in the plan [21].

The HRH costing objectives were: (a) to identify current budget ceilings, and to calculate what policies were financially feasible within the existing resource envelope; (b) to calculate current training costs; (c) to identify the financial implications of scaling-up for each funding source, and; (c) to act as a catalyst in the elaboration of the main plan, and in raising funds [22].

The MoH's Vice-Minister and a senior external consultant were the key leaders of the HRDP exercise, assuming the responsibility of mobilising resources for the elaboration of the plan, and of advocating for the importance of HRH costing as a policy tool. Within the MoH, the Human Resources Department acted as the focal point, providing linkage between consultants and other actors, as well as punctual input in the HRH costing. The MoH Financial Department provided financial information coming from the Ministry of Finance's Budget Department. Personnel of the *Faculdade de Medicina Raul Diaz Arguellez *Medicine School and of the National School of Health were key informants for the identification of training costs. Representatives of aid agencies were supportive but sceptical as to the chances of success of the process of elaboration of an HRH strategy for the country.

The final costing exercise was generally well received by the MoH, although little contributions were made to the documents, as the matter was considered too technical for the human resource department. Shortly after, the final HRDP was elaborated, and both the HRDP and its costing have now been formally adopted by the MoH, although not yet formally approved by the Council of Ministers.

#### Methodology used in Guinea Bissau

Cost projections were based on targets already defined for the HRDP. Personnel projections took into account attrition rates due to deaths and retirement. In a context of scarcity of information on labour market demand and supply, bottom-up and step-down costing were used; the former was employed more frequently, as the costs of most training and funding activities had to be calculated.

Only recurrent expenditures were considered for pre-service and on-the-job training (elsewhere referred to as "Continuous Medical Education"), excluding capital and investment costs, as the focus of the exercise was on the financial feasibility of training and recruiting a minimum number of staff; training facilities investment plans were discussed separately [23]. The assumption was made that the public sector would be able to absorb all new staff trained, with no losses to the private sector or to emigration. Only external funds under the direct control of the MoH were considered as external funds available to finance the HRDP (also known as 'funds for the implementation of the second national health sector development plan'). These included grants/loans from the World Bank, the African Development Bank and the Global Fund for Aids, Malaria and Tuberculosis. Since the majority of donors' funds for health were fragmented in numerous in-house-managed projects, information on a significant share of external funds for HRH training could not be included in the study.

Steps taken to calculate the financial resources needed to train and contract new health personnel were the following: (1) projecting the HRH needs to achieve minimum staff requirements in the public sector for the next 10 years; (2) calculating unit costs of training for each type of personnel; (3) budgeting the salary bill for each year of planning; (4) forecasting availability of internal and external funds, and; (5) constructing an expenditures and funds database and modelling financing scenarios.

HRH needs were available from a previous assessment of HRH on the field [24]. The number of training courses needed to produce the required output was calculated working bottom-up with the financial and planning officers from the local National School of Health, and from the Faculty of Medicine. For courses offered in the past (nurses, midwifes, physicians, laboratory and pharmacy technicians), cost data were extracted from balance sheets and funding documents. For courses not yet available (specialists, anaesthesia and surgery technicians), the monetary value of grants to support studies abroad were calculated with the help of those aid agencies offering such grants (WHO, the Cuban Embassy and Portuguese Cooperation). The inputs for training were: teacher salaries and allowances, student transport and living allowances, books, equipment and equipment maintenance.

**Figure 3:**
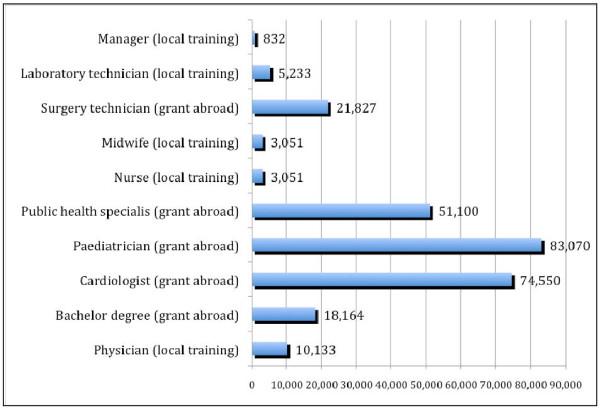
**Training cost for specific HRH in/for Guinea Bissau (2009 US$ constant prices)**. Source: Russo and Ferrinho (2009) 13.

Figure 3 shows that, generally speaking, training professionals locally in Guinea Bissau is cheaper than training them abroad, with physicians trained by the Cuban brigade at an overall cost of US$ 10 133 each for a 5-year course (the Cuban Embassy currently supports the Guinean health sector through a brigade of general practitioners and specialists, who, besides working in the local central hospital, teach at the *Faculdade de Medicina Raul Diaz Arguellez *Medical School). Physicians with a Guinean medical degree going abroad (to Portugal) to specialize in paediatrics were the most expensive category to train, with a cost of US$ 83 070 for a 6-year course in paediatrics.

On-the-job training, an activity not currently carried out, was calculated as representing five days of training per year for existing and future staff (excluding auxiliaries). Cost inputs considered were: teacher remuneration, transport, trainee living allowances and teaching equipment.

Data on basic salary and subsidies (for work in remote areas and extra hours) were obtained from the Ministry of Planning and Finance, and matched with previous years state budgets for health personnel [25]. State budget and external funds financing ceilings were projected from historical expenditures and funds available through the MoH National Health Plan Financing and Implementation Unit.

An expenditure and financing database was built in Excel, including variables on type and number of personnel to be trained and contracted each year, total expenditure, source of funding, category of personnel, and type of expenditure. Through pivot table reports, the database allowed for modelling two expenditure and financing scenarios: one calculated the financial burden for state and donors of training and contracting all the HRHs required to achieve the minimum staff in all NHS facilities. The second scenario calculated the financial implications and funding gaps of a hypothetical salary policy raising by 25% the remuneration of specialised personnel, and by 10% that of mid-level. Both scenarios were compared to projected available state and external funds, and funding gaps were estimated.

Costing and expenditure projections were done in constant base-year prices, as well as in current prices until 2017, taking into account the forecasted inflation rate (according to the formula: Future value = Present value* (1+i)^n^, with the rationale of providing the MoH with an updated tool to negotiate year-on-year future personnel funding needs.

**Figure 4:**
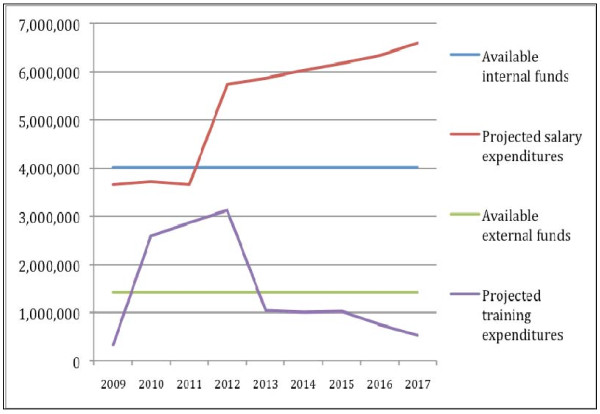
**Guinea Bissau HRH plan's projected expenditures and funding (2009 US$)**. Source: Russo and Ferrinho 2009 13.

The base scenario (Scenario 1) demonstrated that a considerable gap existed between the cost of the full plan and available funds, particularly with respect to the state funds needed to contract new health workers. The projections suggested that in order to close that gap, State funds were required to grow by 6.4% annually in real terms (Figure 4).

As for external funds, an issue of year-on-year availability was identified, rather than one of absolute scarcity, as training expenditures were anticipated to cumulate during the first few years, leaving a financing gap that could be closed only in 2012 (Figure 4).

### Differences and similarities between the costing exercises in Mozambique and Guinea Bissau

The two HRDP costing exercises presented above showed key similarities and differences worth highlighting in order to draw lessons for the wider debate on HRH costing methodologies (Additional file 1: Table S1). First, although apparently similar, the two countries' contexts were uncovered to be remarkably different. On the one hand, Mozambique displayed the characteristics of a post-conflict country, with an emerging post-war middle class, donor aid focussing on the health sector, and an explicit government commitment towards 'social' sectors. On the other hand, at the time of the costing exercise Guinea Bissau was still seen as deeply embroiled in political instability, with a looming HRH crisis, and with limited donor interest in financing an HRH development plan.

In both case studies, costing was conceived as part of a larger planning and fund-raising effort, which in Mozambique was chiefly aimed at extracting salary concessions from the Government's MPF and additional pledges from the international community, while in Guinea Bissau the exercise's focus was more on drawing attention to the health sector's impending human resources crises. Given the declared lack of local health economists and planners, both exercises were developed by a team of international experts with limited participation from local staff.

In both cases, a bottom-up approach was selected to carry out the costing, given the scarcity of information on training unit cost and financial data on the sector's human resources. Simple Excel-based models were designed specifically for both exercises, either creating multiple linked sheets, or a database with respective pivot tables. Both exercises considered mostly basic human resources spending areas, such as initial and on-the-job training, as well as grants to train abroad. In Mozambique, the methodology also included the investment costs of expanding the physical training network. This was not possible in Guinea Bissau as, at the time of the costing exercise, strategic decisions on the final configuration of the facilities network were still pending. Salaries, subsidies, training equipment, and scholarships were the cost categories covered in both exercises.

In both Mozambique and Guinea Bissau, the concluding spending and financing scenarios were designed to evaluate the fiscal impact of HRH policies of interest. For Mozambique, such scenarios were chiefly used to highlight the wide funding gap between identified needs and available resources, with the objective to make an explicit call for extra funds. In Guinea Bissau, the chosen scenarios modelling selected HRH policies and respective funding needs were to some extent more conservative, due to the country's less favourable financing environment and limited training capacity.

## Discussion

This study's methodology, used to compare the Mozambican and Guinean experiences, presents a number of limitations. First, as the two case studies are based on previous work, a policy analysis framework was only applied retrospectively. As the study methods were not applied to design the original fieldwork, but only to interpret its results, the depth and internal validity of our analysis may be limited. Secondly, due to their Portuguese cultural heritage and country's current political situation, the Mozambican and Guinean health systems present very specific features, which may limit the generalisation of the study findings to other contexts. Despite these limitations, a number of lessons from the case studies can be considered valid and possibly applicable to other contexts.

The study primarily shows that costing is part of the more general HRDP process, not a self-standing economic exercise. In Mozambique and Guinea Bissau, HRH costing had multiple objectives: (a) to contribute to extra-health agendas such as public sector salaries, subsidies and incentives policies; (b) to identify wage inequalities; (c) to engage government and external partners in a debate on resources needed for HRH development; (d) to create consensus on the HRDP's validity and relevance; (e) to provide a long-term vision of resource needs for the Ministries of Health; (f) to help define HRH policies, providing MoH managers with essential financial information, and; (g) to help raise funds from the international community. All in all, bringing costs and financial implications into the HRDP equation helped to avoid overambitious plans for workforce expansion, and to show that current spending commitments were insufficient. This is consistent with calls from many scholars for HRH planning to be 'strategic' [1].

The analysis of the two case studies also suggests that the costing methodology needs to be simple and flexible, to cover basic spending areas, and to allow for the evaluation of different policy scenarios' financial and political feasibility. The majority of data on external funds and training input costs were not readily available, and had to be calculated. A significant amount of time had to be spent with local financial officers to interpret financial reporting systems and to identify adequate cost apportioning methods. The implication of this is that the two methodologies had to resort to both top down and bottom up accounting, which allow for more reliable, if time-consuming, cost calculations [26,27]. Furthermore, only the basic areas of wage bills, initial and on-the-job-training (elsewhere referred to as 'continuous medical education'), and capital investment were considered. Restricting the expenditure areas considerably simplified the task, but also made possible the evaluation of alternative policy scenarios, such as increasing/decreasing the number of personnel, increasing training capacity and raising or decompressing salaries under different funding ceilings.

The present study intends to contribute to the debate on resource requirements tools (RRT) for HRH planning. Our study supports the WHO approach of developing a standardised costing approach, in order to reduce local governments' dependency on international experts [5]. However, by highlighting the importance of costing in the general HRDP elaboration process, this study also raises doubts on the opportunity of limiting the task to entering cost data in standard software.

The lack of flexibility of the RRT is called into question, especially with regard to the categorisation of incentive payments and allowances. The RRT's inability to interpret and adapt to local financial situations may jeopardise the whole HRDP process, since the understanding of a country's flows of funds and respective financial information system is what lends the exercise the credibility to achieve its policy goals. Furthermore, the RRT appears at times unnecessarily complicated, bringing into the exercise complex issues such as the private sector health workforce, but dealing only partially with the overall national demand of human resources and health workers' dual practice.

If priority is to be given to developing an approach tailored to each context, regional and international comparisons may not be easy to carry out, and a significant amount of resources may have to be devoted to costing exercises. However, such types of investment may be required to address the health workforce crisis effectively and to design comprehensive and long-term solutions.

## Conclusions

This study compared two separate costing exercises conducted in Mozambique and Guinea Bissau, with the objective to contribute to the existing debate on HRH costing methodologies. The adoption of a policy-oriented analysis framework implied that the two countries' policy contexts, processes and actors involved were factored into the analysis of the methodologies developed.

We conclude that a HRDP costing methodology needs to be integrated in the general HRH plan elaboration process, to be simple and flexible and as country-specific as possible. While recognising the value of designing generic approaches and tools, the case studies show that a successful HRH costing methodology is a balancing act between simplicity, relevance and comprehensiveness, which may be difficult to achieve through a standardised software-based approach.

More research and case study analysis appears to be needed to understand how HRH costing can be turned into simple, standardised, policy-supportive exercises, performed by national teams, without losing its country-specificity and relevance.

## Competing interests

The authors declare that they have no competing interests.

## Authors' contributions

AKT carried out part of the fieldwork, participated in the study conception and in drafting the manuscript. GR participated in the design of the study, carried out part of the fieldwork, drafted and edited the manuscript. GD participated in the study conception and design, and in drafting and editing the manuscript. PF participated in the fieldwork and in editing the manuscript. All authors read and approved the final manuscript.

## Supplementary Material

Additional file 1 **Table S1**. Cost components and sub-components of the HRH costing strategies developed in Mozambique and Guinea Bissau Click here for file
