# Supplementary material for: Costing the scaling-up of human resources for health: lessons from Mozambique and Guinea Bissau
Source: Hum Resour Health. 2010 Jun 25;8:14. doi: 10.1186/1478-4491-8-14 (PMC2902408; doi:10.1186/1478-4491-8-14)
Supplement: Additional file 1 — Table S1. Cost components and sub-components of the HRH costing strategies developed in Mozambique and Guinea Bissau [file 1478-4491-8-14-S1.DOC]

Cost components and sub-components of the HRH costing strategies developed in Mozambique and Guinea Bissau

| Component | Sub-component | Notes and assumptions in Mozambican methodology | Notes and assumptions in Guinean methodology |
| --- | --- | --- | --- |
| 1.Salaries and allowances (Wage Bill) | Base salaries | Drawn from government salary scales (Ministry of Finance) and presented per level and profession or category of personnel. | Drawn from MINFIN salary tables, and presented per profession, level and category of personnel. |
|  | Allowances | Calculated as a percentage of base salaries, which varies depending on the level and profession or category of personnel. | Two types of allowances were considered (night shift and rural residence subsidies), calculated as proportions of base salaries. |
| 2. Incentives package | All existing financial and non financial incentives, not included in allowances above | Calculated as a percentage of the wage bill, assuming the percentage calculated in 2006 remains constant. | Productivity incentives do not currently exist in Guinea Bissau. |
| 3. Training | Pre-service training | Includes fixed and variable cost of pre-service training, cost of central level purchases, teacher training, expanding and upgrading the training network, contracting of private services. Costs based on expenditure analysis of training institutions. Variable cost calculated from unit cost per student per level and course specialty and total number of students. | Costs calculated bottom-up separately for Training Hospital and National School, on the basis of past courses. These included: teacher salaries and allowances, student transport and living allowances, books, equipment and equipment maintenance. |
|  | In-service training | Number of beneficiaries based on training to date and prioritized specialties. Calculation relies on unit cost per person per type of course. Total cost based on number of beneficiaries for each type of course. Also considers cost of strengthening in-service training departments at all levels. | On the job training currently not existent in Guinea Bissau. Hypothetical costs were calculated for all health personnel excluding auxiliaries, and included teacher salaries and allowances, student transport and living allowances, books, equipment and equipment maintenance. |
|  | Post-graduate training | Includes post-graduate training abroad, as well as the cost of running post-graduate courses in Mozambique (specialist doctors in particular). | Calculated on the basis of past scholarships offered to MISAP cadres by WHO and UNICEF. |
|  | Scholarships | Includes scholarships in Mozambique and abroad. Number of beneficiaries estimated as a percentage of workforce, with the assumption that actual percentage of beneficiaries remains constant. | Calculated on the basis of past scholarships offered to MISAP cadres by WHO and UNICEF for specific countries. |
| 4. Human resources management (HRM) strengthening | In-service training of HR managers | Average cost per manager trained per course (based on unit cost of in-service training calculated), timed by number of students per course times total number of courses. | No such policy included in the Guinean HRDP. |
|  | Setting up of national health management school | Initial capital investment and recurrent expenditure. | No such policy included in the Guinean HRDP. |
|  | Decentralization of HR management to provincial and district level | Cost of equipment and necessary materials. | No such policy included in the Guinean HRDP. |
|  | Development and implementation of observatory for HRM | In large part cost of meetings and seminars. | No such policy included in the Guinean HSDP. |
| 5. Foreign doctors | Specialists and general practitioners | Total number of foreign doctors corresponded to total needs estimated minus national production capacity on an annual basis. | Costs of Cuban doctors calculated bottom-up with the *Faculdade Raul Arguellez* Cuban brigade. |
| 6. Community Health Workers (CHW) | Allowances | Cost calculation made based on 3000 CHWs to be trained and deployed. | No such category is currently trained nor employed in Guinea Bissau. |
|  | Benefits in cash and kind |  |  |
|  | Training |  |  |
|  | Equipment/materials and drug kits |  |  |
|  | Supervision |  |  |
| 7.Facilitation of the plan’s implementation | Creation of a task force | Costs associated with national, regional and international travel, as well as for the organization of seminars, workshops, etc. | Personnel, goods and services costs were considered, associated with strengthening MoH capacity. |
|  | Local and international technical assistance | To support MoH in design of further strategies (e.g. for in-service training, incentives policy, etc.), and advisory role to the task force. | Strategy not included in the costing. |
| Health system | Infra-structure | Based on health network upgrading and expansion planned by MoH | Only running costs considered. |
|  | Equipment | Based on recurrent expenditure projection of existing costing of Health Sector Strategic Plan (excluding personnel costs) | Only basic equipment for training was considered in the costing. |
